# Extracellular Vesicle Transportation and Uptake by Recipient Cells: A Critical Process to Regulate Human Diseases

**DOI:** 10.3390/pr9020273

**Published:** 2021-01-31

**Authors:** Zhi Hao Kwok, Chenghao Wang, Yang Jin

**Affiliations:** Division of Pulmonary and Critical Care Medicine, Department of Medicine, Boston University Medical Campus, Boston, MA 02118, USA;

**Keywords:** extracellular vesicles, uptake specificity, endocytosis

## Abstract

Emerging evidence highlights the relevance of extracellular vesicles (EVs) in modulating human diseases including but not limited to cancer, inflammation, and neurological disorders. EVs can be found in almost all types of human body fluids, suggesting that their trafficking may allow for their targeting to remote recipient cells. While molecular processes underlying EV biogenesis and secretion are increasingly elucidated, mechanisms governing EV transportation, target finding and binding, as well as uptake into recipient cells remain to be characterized. Understanding the specificity of EV transport and uptake is critical to facilitating the development of EVs as valuable diagnostics and therapeutics. In this mini review, we focus on EV uptake mechanisms and specificities, as well as their implications in human diseases.

## 1. Introduction

Extracellular vesicles (EVs) are heterogenous, membrane-bound packages containing complex cargos including nucleic acids, lipids, and proteins. While EVs were initially considered to be mechanisms for the discharge of cellular wastes [1], increasing evidence has implicated EVs as an important mean of intercellular communication via the transference of their cargo contents between cells [2–4]. Over the years, EVs have been broadly classified into two categories, namely exosomes and microvesicles (MVs), according to their physical sizes, biogenesis pathways, and cell surface markers. MVs are produced by the outward budding followed by pinching of the plasma membrane and range from 100 nm to 1000 nm in diameter [5]. In contrast, exosomes typically range from 30 nm to 100 nm in diameter and are formed as multivesicular endosomes (MVEs) from the maturation of intraluminal vesicles (ILVs), prior to their secretion via fusion with the cell membrane [6]. Owing to the overlap in sizes, as well as the lack of consensus on specific surface markers of these EV categories, the International Society of Extracellular Vesicles (ISEV) has suspended and highly discourages the use of the aforementioned nomenclature for EV classification. Instead, the current guidelines set by ISEV follows that EVs can be termed based on, “(a) physical characteristics of EVs, such as size (“small EVs” (sEVs) and “medium/large EVs” (m/lEVs), with ranges defined, for instance, respectively, <100 nm or <200 nm [small], or >200 nm [large and/or medium]) or density (low, middle, high, with each range defined); (b) biochemical composition (CD63+/CD81+- EVs, Annexin A5-stained EVs, etc.); or (c) descriptions of conditions or cell of origin (podocyte EVs, hypoxic EVs, large oncosomes, apoptotic bodies)” [7,8].

As sEVs (primarily the MVs and exosomes in the previous nomenclature) are implicated to a greater extent in various human diseases, we will focus on this EV subgroup throughout this review, unless otherwise stated.

EVs can be detected in almost all body fluids—including saliva, tears, blood, urine, and semen—and increasing evidence has pointed to its critical roles in physiological processes such as angiogenesis and immune regulation [9–11], as well as pathological conditions including neurological diseases and cancer [12,13]. EV uptake into recipient cells and the subsequent release of its contents—comprising of functionally active RNAs (such as microRNAs (miRNAs), mRNAs, and long non-coding RNAs (lncRNAs) and proteins—can modulate gene expression through the post-transcriptional regulation of target mRNAs and de-novo translation of EV-derived mRNAs [14]. Alternatively, EVs can induce intracellular signaling pathways in recipient cells via surface ligand-receptor interactions. Such alterations in gene expression and deregulation of signaling activities within the cells may result in phenotypic changes, leading to disease onset and progression.

The unique abilities of EVs to protect their cargo from enzymatic degradation and be modified for specific cell-targeting have also garnered massive interests for their potential as natural delivery vectors for therapeutic molecules. With emerging functions in physiological and pathological conditions, as well as therapeutic potential, it is imperative to understand the molecular mechanisms governing EV uptake by recipient cells. In this mini review, we will summarize the different ways in which EVs enter target cells and review the current knowledge on the specificity of EV uptake. In addition, we will highlight the roles of EVs in human diseases and discuss the potential of EVs as diagnostic and therapeutic agents for clinical applications.

## 2. EV Biogenesis, Isolation, and Characterization

Biogenesis of EVs mainly involves (1) the outward budding followed by pinching of the plasma membrane (commonly employed by MVs) and (2) the formation of multivesicular endosomes (MVEs) from the maturation of intraluminal vesicles (ILVs) (commonly employed by exosomes). MV shedding can often induced by intracellular physical and chemical activation such as an increase in cytosolic Ca^2+^ levels, as well as apoptosis [15]. MV formation can also be induced by the activation of the RHO family of small GTPases and RHO-associated protein kinase (ROCK), key regulators of actin assembly and disassembly [16].

**Figure 1.**
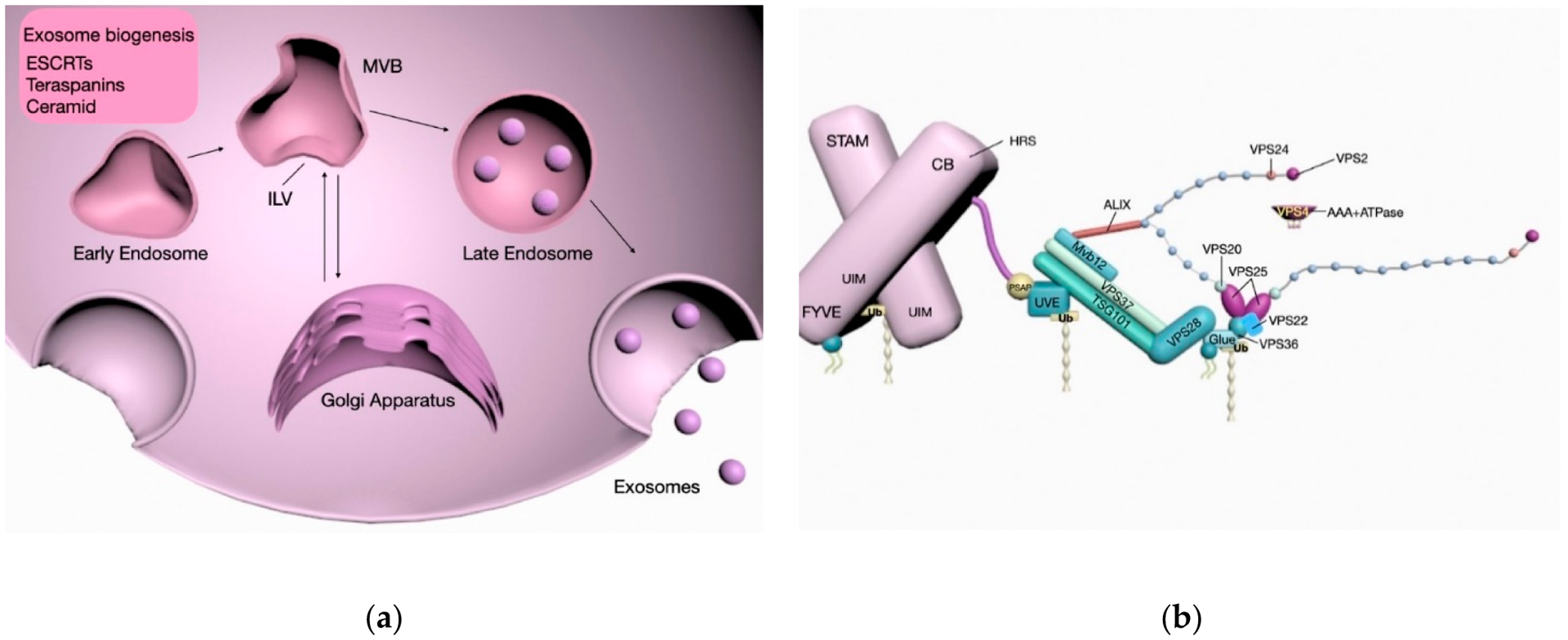
Schema of the molecular mechanisms and interactions involved in exosome biogenesis. Exosomes can be formed as intraluminal vesicles (ILVs) in an endosomal sorting complex required for transport (ESCRT)-dependent manner, as well as via ESCRT-independent pathways involving molecules such as ceramide, tetraspanins, and proteoglycans prior to their secretion (**a**). Post-translational modifications of the vacuolar protein sorting-associated proteins (VPS) family proteins involved in the sorting of exosomal cargo and generation. Energy required for the scission of budding exsomes from membrane is dependent on the ATPase activity of the Vacuolar protein 4 (VPS4) complex (**b**).

As opposed to MV biogenesis at the plasma membrane, exosomes originate from the endosomal compartment and involve multiple mechanisms that are responsible for processes ranging from cargo sorting to the transport and apposition of MVEs at the cell membrane for their release. The molecular processes mediating exosome formation can generally be distinguished by the involvement of the endosomal sorting complex required for transport (ESCRT) machinery (Figure 1).

### 2.1. ESCRT-Dependent Biogenesis

The involvement of ESCRT machinery in membrane shaping and scission provided insights into the mechanisms underlying ILVs and MVEs formation. It is now well established that the ESCRT-0 and ESCRT-I subunits form stable hetero-oligomers that act to recognize and cluster ubiquitinated cargo proteins, and thereafter recruit ESCRT-II for the assembly of the ESCRT-III complex to mediate membrane budding and scission [17].

### 2.2. ESCRT-Independent Biogenesis

Alternatively, exosomes can be generated in an ESCRT-independent manner involving ceramide, the syndecan/ALIX pathway and tetraspanins [18–20].

Although these mechanisms may be molecularly distinct from one another, exosome biogenesis often involves the concomitant dependence on multiple ESCRT-dependent and ESCRT-independent pathways governed by factors including cargo content, cell type, and external stimuli. Moreover, these different ways of production may account for the heterogeneity observed in EV populations secreted by different cells.

### 2.3. EV Cargo

It is well established that EVs carry a plethora of molecules, ranging from DNAs, RNAs to proteins and lipids, which can be transferred to recipient cells to elicit functional effects. However, little is known about the underlying mechanisms that may account for the specific repertoires of EV cargo as well as the heterogeneity in cargo compositions across different EVs populations and subtypes. Increasing evidence has pointed to the selectivity in cargo loading during EV biogenesis. Rather than a ‘universal’ regulation of cargo sorting into EVs, studies have demonstrated that it is a highly selective process that may be influenced by factors such as cell type of origin, physiological status of the donor cells and external stimulation [21,22]. Furthermore, a multitude of studies have identified specific proteins that may mediate the selective loading of molecules into EVs [23–26]. Critically, post-translational modifications of these proteins are increasingly implicated in modulating their functions and the consequent sorting of their bound molecules into EVs [27].

### 2.4. MVE Secretion

Prior to their secretion into the extracellular space, MVEs must be transported, docked, and fused with the cell membrane. In general, the intracellular trafficking of MVEs requires molecular motors such as myosin, kinesins and dynein as well as GTPases for the association with and rearrangement of the dynamic cytoskeleton. For example, the Rab11 and Rab35 proteins have been shown to affect the docking and fusion of MVEs in erythroleukemia cells and oligodendrocytes, respectively [28,29]. In addition, Rab27a and Rab27b isoforms were demonstrated to positively regulate the motility of MVEs and docking at the plasma membrane via synaptotagmin-like protein 4 and exophilin 5 effector proteins [30]. Much like biogenesis, processes and the associated molecular regulators facilitating MVE secretion tend to vary across cell types and may be affected by the exposure to different exogenous stimuli.

### 2.5. EV Half-Life

Intravenously administered EVs were found to be detected as early as 2 min and can remain detected for as long as 30 min [31–33]. Studies have indicated that the in vivo half-life, biodistribution and clearance of EVs can vary greatly depending on factors including route of administration, cell-type origin, and availability of target cells for EV internalization [34].

### 2.6. EV Separation and Characterization

Biophysical and biochemical properties such as size, density, morphology, charge, and presence of different surface antigens can allow for the differentiation between MVs from exosomes or other EV subtypes. Based on the differences in these variables, EV isolation methods commonly include differential centrifugation, density gradient centrifugation, ultrafiltration, size exclusion chromatography, and immunoprecipitation assays [35].

However, isolation and purification by the various methods as described above is insufficient to accurately classify vesicles as exosomes or microvesicles. Instead, a combination of quantitative (such as protein composition) and qualitative (such as morphology and physical characteristics) criteria is necessary for the precise distinction between the different populations of vesicles. Physical features including size, as well as morphology, can be confirmed by transmission electron microscopy (TEM), which provides direct visualization of the vesicles. Alternatively, the use of nanoparticle tracking analysis (NTA) can enable the determination of vesicle size as well as vesicular concentration [36]. As for the biochemical characterization of vesicles, exosomal surface markers can be identified quantitatively with traditional methods including immunoblotting, flow cytometry, or proteomic profiling by mass spectrometry analysis.

## 3. EV Uptake

Upon their release from donor cells, EVs can interact with recipient cells to induce intracellular signaling and changes to molecular processes that may lead to alterations in their physiological or pathological states, either through binding with surface receptors or internalization and release of their cargo contents. Evidence for EV internalization was provided in multiple studies, including one that demonstrated the direct transfer of mouse RNAs and consequent detection of mouse proteins in human mast cells [37]. EV uptake was further substantiated by studies that showed the successful knockdown of target gene expression and production of bioluminescence via EV-mediated delivery of small interfering RNAs (siRNAs) and luciferin substrates, respectively [38,39].

**Figure 2.**
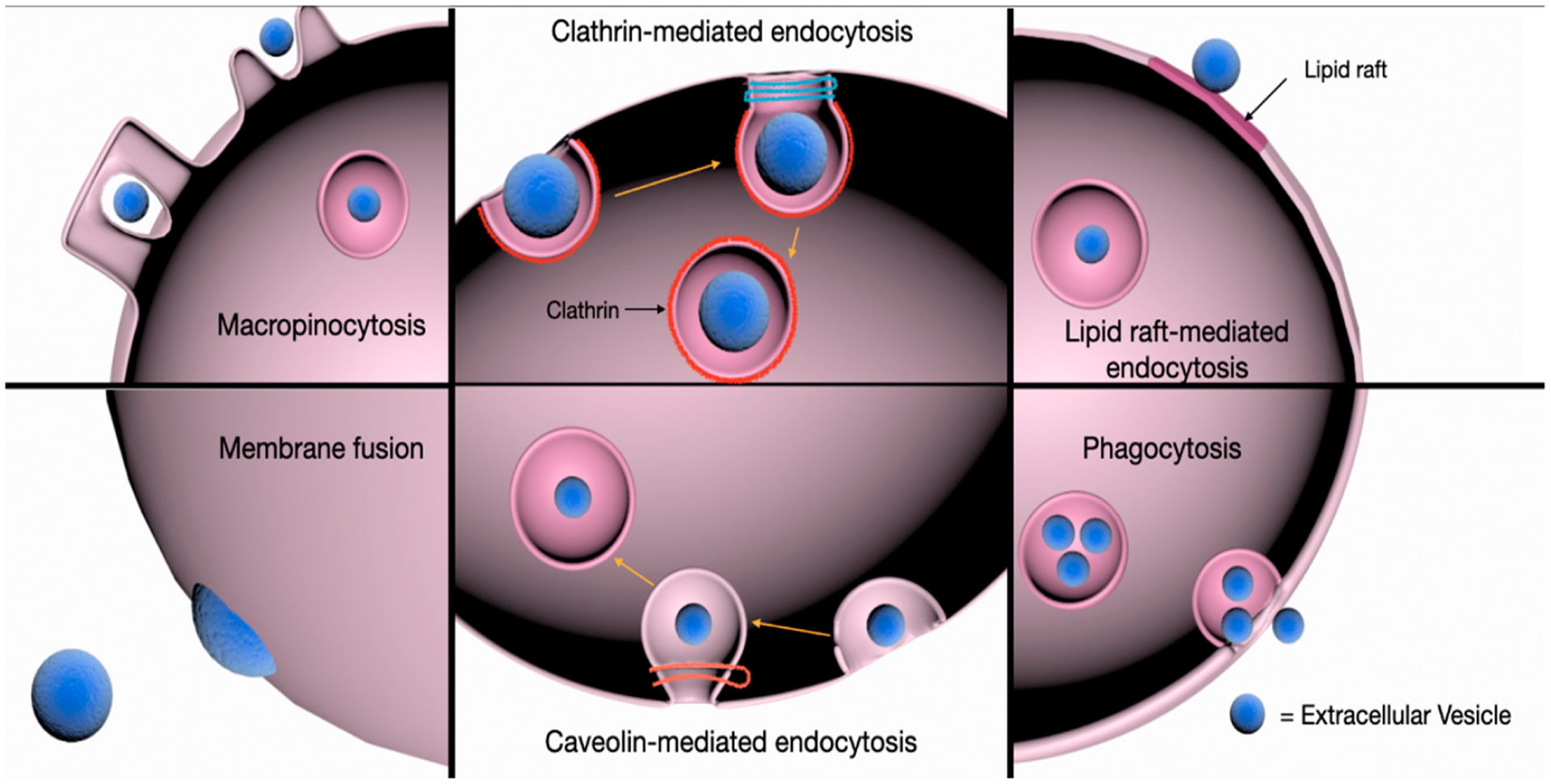
Schema of the pathways involved in EV uptake by recipient cells. EVs can be internalized into target cells via clathrin- and caveolin-mediated endocytosis, phagocytosis, and macropinocytosis. The role of lipid rafts in clathrinand caveolin-dependent and -independent endocytosis of EVs has also been described. Alternatively, EV cargo may be internalized following the direct fusion of EVs with the plasma membrane of target cells to induce phenotypic responses.

To date, majority of the experimental evidence indicates that EVs are typically internalized into the endosomal compartment by endocytosis [40]. However, the exact mechanisms governing the endocytosis of EVs remain highly debatable. Various mechanisms have been proposed, including clathrin-mediated endocytosis (CME), caveolin-dependent endocytosis (CDE), micropinocytosis, and phagocytosis (Figure 2). Additionally, the relevance of lipid raft proteins and specific protein–protein interactions in EV internalization have also been illustrated. Generally, the docking and subsequent endocytosis of EVs is facilitated by protein–protein interactions with membrane receptors, ligands or contact proteins of recipient cells. Proteins such as tetraspanins, lectins, proteoglycans, and integrins, as well as their PTMs have been implicated in these specific interactions to affect EV uptake.

### 3.1. Tetraspanins

Tetraspanins are membrane proteins that are abundantly found on the EV surfaces and known to be involved in cell adhesion and signaling [41]. The formation of tetraspanin-enriched microdomains (TEMs), clusters comprising of tetraspanins, adhesion proteins, and transmembrane receptors at the plasma membrane mediates vesicular fusions and plays a role in EV docking and uptake [20]. Their regulatory role in EV internalization was further substantiated by the reduced EV uptake into dendritic cells after the inhibition of tetraspanin CD9 and CD81 [42]. In addition, EVs containing the Tspan8-CD49d complex on their surfaces were shown to be readily internalized by endothelial and pancreatic cells, presumably due to interactions with the intracellular adhesion molecule (ICAM-1) present on the membrane surfaces of these cells [43].

### 3.2. Lectins and Proteoglycans

Lectins such as DC-SIGN and DEC-205 were similarly found to be involved in EV binding and uptake; inhibition of EV internalization into monocyte-derived dendritic cells was observed following treatment with specific antibodies targeting these receptors [44,45]. Apart from tetraspanins and lectins, proteoglycans, proteins that are heavily glycosylated with one or more covalently attached glycosaminoglycan (GAG) chains, have also been implicated in EV binding and uptake. For instance, Glypican 1, a heparan sulphate containing proteoglycans was shown to be highly enriched in cancer cell-derived exosomes and mediate their attachment to recipient cells [46]. Furthermore, modifications of the glycosylation profiles of EV surface proteoglycans were found to affect the affinity for EVs by a variety of tested cell lines [47].

### 3.3. Integrins

Integrins, known for their functions—such as cell-to-cell adhesion, cell signaling, and leukocyte migration—have also been reported to play key roles in EV docking and internalization. For example, the role of integrin avβ3 in the adhesion and uptake of sEVs by breast cancer cells was demonstrated when sEV uptake was significantly inhibited upon its blockade with a disintegrin inhibitor (Dis*Ba*-01) [48]. Similarly, integrin beta 3 (ITGB3) was reported to play a central role in the recognition of heparan sulfate proteoglycans (HSPGs)-associated EVs and subsequent focal adhesion kinase (FAK)-mediated endocytosis of these vesicles [49]. Furthermore, integrin composition and their consequent heterodimerization on surfaces of cancer cell-derived exosomes was shown to affect their tissue-specific targeting to the lungs and liver [50].

### 3.4. Clathrin-Mediated Endocytosis (CME)

CME is an active process in which EVs can be internalized via the sequential formation of clathrin-coated vesicles which contain a variety of transmembrane receptors and small ligands. The assembly of these clathrin-coated vesicles progressively deform the membrane, leading to its collapse into a vesicular bud which then matures and pinches off from the cell surface. Clathrin proteins are subsequently uncoated from the internalized vesicle, allowing it to fuse with the endosome for the release of its contents [51]. Treatment with chlorpromazine, which prevents the generation of clathrin-coated pits at the cell membrane, inhibited EV uptake by phagocytic cells and ovarian cancer cells respectively [52,53]. Importantly, the siRNA-mediated depletion of the clathrin heavy chain (CHC) inhibited EV internalization [54], indicating that CME is at least in part involved in EV uptake.

### 3.5. Caveolin-Dependent Endocytosis (CDE)

Similar to CME, CDE involves the formation of small, cave-like invaginations known as caveolar vesicles within the plasma membrane that are eventually pinched off and internalized. Caveolae are domains of glycolipid rafts in the cell membrane that are rich in caveolins, cholesterol and sphingolipids. The formation of caveolae requires the caveolin proteins, whereby the oligomerization of these caveolins via the caveolin oligomerization domains mediates the generation and assembly of caveolin-rich rafts within the plasma membrane [55]. Caveolin-1 (Cav-1) protein alone was found to be sufficient for inducing caveolae formation and the specific knockdown of Cav-1 expression resulted in a significant impairment of EV uptake [56]. In another study employing an ischemia and reperfusion injury (IRI) mouse model, neuronal cells were shown to actively upregulate Cav-1 expression to enhance the uptake of human umbilical vein endothelial cell(HUVEC)-derived EVs, which could confer cytoprotective effects for their survival [57].

### 3.6. Macropinocytosis

A process that is commonly referred to as ‘cell drinking’, uptake via macropinocytosis entails the generation of invaginated membrane ruffles and the subsequent pinching off into the intracellular space. The protrusion of ruffled extensions of the plasma membrane allows molecules or EVs to be ‘trapped’ and subsequently internalized upon the fusion of these protrusions, either with the plasma membrane or themselves [58]. Macropinocytosis is dependent on the activity of the Na^+^/H^+^ exchanger and requires cholesterol for the recruitment of activated rac1 GTPase to restructure the actin cytoskeleton at the sites of invagination [59,60]. Blocking macropinocytosis via the inhibition of the Na^+^/H^+^ exchanger and rac1 resulted in decreased oligodendrocyte-derived EV uptake in microglia cells [61], highlighting the role of this process in EV internalization.

### 3.7. Phagocytosis

As opposed to macropinocytosis, phagocytosis is a receptor-mediated process that does not involve direct contact with the internalized molecules, nor require the extension of membrane ruffles [62]. Otherwise, phagocytosis similarly requires the sequential formation of membrane invaginations encompassing the material to be taken up [62]. Although primarily utilized by macrophages for the engulfment of particles such as bacteria and apoptotic fragments, phagocytosis has been identified as an efficient uptake mechanism for EVs [52]. The role of phagocytosis in EV uptake was further substantiated when the inhibition of PI3K by LY294002 and wortmannin, a kinase that is involved in the process of membrane insertion for the formation of phagosomes [63], led to a dose-dependent reduction in EV uptake [52].

### 3.8. Involvement of Lipid Rafts

Lipid rafts are transient and highly dynamic microdomains in the plasma membrane with an abundance of phospholipids, cholesterol, sphingolipids, and glycosylphosphatidylinositol (GPI)-anchored proteins [64]. Owing to the distinct physical properties attributed to the varied composition of lipid rafts, they can act as scaffolds for the recruitment and assembly of signaling complexes to affect membrane fluidity and protein trafficking [65]. Lipid rafts can be found in the clathrin and caveolar-coated vesicles and thus involved in both clathrin- and caveolin-mediated endocytosis. Alternatively, they can also be localized to flotillin-enriched membrane regions, in which their associations mediate clathrin- and caveolin-independent endocytosis [66–69]. The potential role of lipid rafts in affecting EV uptake was confirmed by studies employing inhibitors of cholesterol and glycosphingolipid synthesis. For example, treatment with fumonisin B1 and N-butyldeoxynojirimycin hydrochloride, compounds known to reduce glycosphingolipid composition in the plasma membrane via blockade of its biosynthesis [70,71], as well as cholesterol reducing agents such as filipin and simvastatin, significantly decreased EV uptake in recipient cells [72–74].

### 3.9. Membrane Fusion

While majority of the research on EV uptake supports a primarily endocytic mechanism, a handful of studies have shown that direct fusion of the EVs and plasma membrane is a possible route for EV internalization and release of its contents [39,74]. Direct contact between the two lipid bilayers in proximity generates a fusion stalk which further expands into a diaphragm bilayer, allowing the formation of a pore whereby the two hydrophobic cores are mixed [75,76]. Proteins that are known to be involved in membrane fusion include the family of the SNARE proteins [77,78] and Sec1/Munc-18 related proteins (SM-proteins) [79]. By employing fluorescent lipid dequenching techniques, EVs were observed to fuse with the plasma membranes of recipient melanoma cells, and fusion was enhanced under acidic [74].

## 4. EV Transportation and Uptake: Specific or Random?

Despite advances in our understanding of the molecular mechanisms underlying EV internalization, the long-standing question of EV uptake specificity remains to be comprehensively addressed in the field. While studies have indicated the prevalence of EV uptake into any cell type tested [73,80], results from other studies demonstrated that EV uptake is a highly specific process in which the recipient cells and EVs would require the ‘right’ type of surface receptors and ligands for the coordinated protein–protein interactions [43,50,80–84]. For example, the fusion of anti-epidermal growth factor receptor (EGFR) nanobodies to glycosylphosphatidylinositol (GPI) anchor signal peptides on the EV surface was sufficient to alter their cell targeting behavior and promote efficient binding to tumor cells that are dependent on EGFR density [81]. In addition, CD63-positive EVs were specifically bound to neuronal and glial cells, whereas CD63-negative EVs targeted only to the dendritic cells of neurons [82]. Recent evidence also indicated that the transfer of secreted exosomes is selective to the cell type of origin i.e., exosomes are preferentially taken up by cell types where they were originally secreted from [83,84]. Furthermore, EV uptake can also be affected by factors such as metabolic status of the recipient cells, and attributes including the types and characteristics of secreted EVs [47,84]. For example, it was observed that neural stem cells tend to exhibit a significantly higher capacity of internalizing EVs in comparison to mature neurons, implying that metabolically active cells may display higher rates of active EV uptake than terminally differentiated cells [84]. Alternatively, the modifications of EV surface glycosylation patterns, leading to either changes in glycosylation states or vesicular charges, were found to affect the subsequent uptake of the EVs. Additionally, high-content screening revealed the preferential affinities for EVs with varying surface glycosylation states by different recipient cell types [47]. However, a recent study involving human mesenchymal stem cells (HSMCs)-derived EVs demonstrated a common HSPG-sensitive and caveolin-mediated endocytic uptake independent of lineage-specificity of the donor HSMCs and recipient cell types [85]. A caveat on these existing data is that the heterogeneity in the populations of donor/recipient cells and EVs may confound some of the observations and contribute to their discrepancies.

Taken together, these results provided insights into how cells may selectively govern EV uptake. With the constant development of novel technological methodologies for studying EV uptake [86,87], detailed mechanisms governing this complex process will no doubt be elucidated in the near future. Nevertheless, it is apparent that a plethora of molecular mechanisms exist to mediate EV-cell communication and different combinations of these mechanisms may be employed by different EV and recipient cell types, depending on inherent properties of EV or dynamic changes in the physiological states of recipient cells.

### Challenges in Studying EV Transportaion and Uptake

Despite the increasing characterization of molecular processes involved in EV uptake, advances in this field are still impeded by many scientific and technical hurdles. For instance, inadequate knowledge of the specific surface markers for different EV classes, coupled with the inherent heterogeneity of EV populations, limit the applicability of employing specific antibodies (to block ligand/receptor interactions), small molecule inhibitors and RNA interference to systematically identify the dominant molecular events that promote EV internalization in various physiological conditions. Secondly, advances in EV transportation research are hindered by the lack of high-throughput technology for the accurate and reliable detection, evaluation and tracking of EVs. For example, developing molecular labeling dyes that are highly stable against cleavage and degradation, together with novel imaging methods, will be useful for in vivo tracing of EVs. In addition, inherent physicochemical properties of EVs, such as a relatively short half-life, limits the time-course monitoring of EV trafficking and uptake.

## 5. EV Uptake in Pathophysiological Diseases

Nevertheless, extensive research in the recent years have clearly demonstrated the critical implications of EV uptake in the pathophysiology of multiple diseases (Table 1). EV surface receptors-stimulated intracellular signaling pathways, as well as trafficking and release of biomaterials, such as nucleic acids and proteins, into recipient cells may deregulate gene expression and disrupt signaling pathways, leading to alterations in the functions and phenotypes of recipient cells. Due to the paramount interests in the emerging roles of EVs in cancer, inflammation and immunity, as well as neurodegeneration, we highlight the implications of EVs in these diseases in this review.

### 5.1. Cancer

A multitude of evidence point towards the increased production and release of EVs with functional alterations in cancer cells as opposed to normal cells, potentially attributed to the elevated expression of genes including the Rab proteins [102], syntenin [103], and heparinase [104]. The altered secretion and cargo composition of tumor EVs can promote tumor growth by affecting the various processes of cancer hallmarks, ranging from apoptosis, invasion, and metastasis to metabolism and tumor microenvironment [105]. For example, the elevated levels of miR-23a and miR-105 in EVs secreted by cancer cells suppressed the expression of prolyl hydroxylases (PHD1/2) and tight junction protein ZO-1, leading to an accumulation of HIF-1α and increased vascular permeability in the surrounding endothelial cells to enhance angiogenesis [89,90]. Additionally, tumor EV-derived RNAs were found to activate the Toll-like receptor 3 (TLR3) in lung epithelial cells, leading to the stimulation of chemokine secretion and increased neutrophil infiltration to favor lung metastasis [91]. Furthermore, EV-mediated transfer of the lncRNA, named lncRNA Activated in RCC with Sunitinib Resistance (lncARSR), functions as a microRNA sponge for sequestering miR-34 and −449, resulting in the increased expression of tyrosine kinases AXL and c-MET to confer sunitinib resistance [106].

### 5.2. Inflammatory Diseases

EVs have also been extensively studied in the context of inflammation and autoimmune diseases, whereby the cargo and surface repertoire of EVs containing immune-related molecules (TGF-β, cytokines), transcriptional factors, as well as enzymes, can exert immunomodulatory effects upon their internalization into recipient cells [107]. For example, EVs secreted from neutrophils were found to mediate vascular inflammation in atheroprone endothelial cells, partly through the miR-155/NF-κB axis, leading to the development of atherosclerosis [94]. In rheumatoid arthritis, T cells and monocytes-derived EVs greatly induced the synthesis of matrix metalloproteinases (MMPs), including MMP-1, -3, -9, and -13, in the recipient fibroblasts and promoted the invasive and destructive phenotype of these cells, leading to the eventual manifestation of degraded bone and cartilage [108].

### 5.3. Neurodegenerative Diseases

A common molecular hallmark of several neurodegenerative diseases is the aggregation of infectious isoforms or misfolded proteins, such as the prion protein (PrP^C^) with its conformational isoform PrP^SC^ in prion disease, β-amyloid plagues in Alzheimer’s disease (AD), and α-synuclein fibrils in Parkinson’s disease (PD). Since these aggregated proteins are often transported in EVs, it is of little surprise that EV uptake may play a role in influencing the progression of these diseases. In AD, decreased EV biogenesis through the inhibition of nSMase2 was demonstrated to reduce plaque formation in vivo and impede disease progression, implicating the disease-stimulating potential of EV cargos in Alzheimer’s [98]. However, EVs were also shown to confer protective effects in AD, potentially via the sequestration of β-amyloid aggregates by the interaction between the PrP^C^ receptors on EV surfaces and the toxic Abeta42 peptides [109,110]. Similarly, the functional role of EVs remains dichotomous in the context of PD. The reduction in exosomal release of α-synuclein, owing to a mutation in the PARK9/ATP13A2 ion pump found in MVEs, was shown to allow the increased clearance of the toxic oligomers and consequently lower intracellular levels of α-synuclein [111,112]. However, studies have also supported the EV-mediated dissemination of toxic α-synuclein oligomers from donor to recipient cells, potentially in a lymphocyte activation gene 3 (LAG3)-dependent manner [113], to accelerate disease progression [100].

### 5.4. Challenges in Studying Functional EV Cargo in Diseases

Despite the increasing identification of functional EV cargos in specific pathological contexts, a comprehensive analysis of these molecules is generally hindered by factors such as EV half-life and the inherent truncation of these EV cargos. Truncated nucleic acids and proteins can result in inconsistencies and unreliable detection of these EV cargos with the currently available sequencing and array platforms. Furthermore, the low copies of these EV cargos limits the ability to accurately quantify their differential expression levels under various physiological and pathological conditions.

## 6. Diagnostic and Therapeutic Applications of EVs

Over the past decades, efforts to develop new therapeutic strategies have focused on the utilization of nanoparticles, including synthetic gold nanoparticles, liposomes and adenovirus, for drug delivery. Amidst the demonstrated delivery efficacies with these nanostructures, the widespread use of these systems has been limited by factors including (1) the inability of these structures to facilitate crossover between different biological barriers and (2) the triggering of undesired inflammatory responses by repeated administration of these particles. In contrast, the intrinsic characteristics of EVs, such as (1) small size to reduce their clearance and allow passive entry into tissues, (2) enclosed membranal structure acts as a protective shield for the encapsulated cargo against degradation during delivery, and (3) modification of the EV cargo and surface proteins allows for the enhanced targeting specificity to certain tissues, making them a highly valuable drug delivery tool option for therapeutic strategies against a variety of pathological diseases.

A multitude of studies have shown promising results of employing EVs to deliver innovative therapies (RNAs, proteins, and therapeutic drug molecules) to specific target cells. For example, loading of curcumin, a highly hydrophobic drug with anti-oxidant and anti-inflammatory properties, into a murine cell line (EL-4)-derived exosomes was demonstrated to effectively decrease the in vitro secretion of inflammatory cytokines, including TNF-α and interleukin-6 (IL-6) by macrophages. In vivo, treatment of lipopolysaccharides (LPS)-induced septic shock mice with curcumin-incorporated exosomes significantly reduced lung inflammation and exhibited increased overall survival [114]. In another study, the delivery of exosomes loaded with anticancer drugs, such as doxorubicin and paclitaxel, across the blood–brain barrier (BBB) was found to exhibit enhanced therapeutic efficacy in a zebrafish brain-cancer model [115]. Apart from small inhibitor molecules and siRNAs, exosomes incorporated with large proteins such as catalase were also efficiently delivered across the BBB to the brain tissue, leading to the neutralization of reactive oxygen species (ROS) and reduced inflammation in a PD mouse model [116]. Indeed, the exploitation of EVs as novel strategies for human diseases are clearly evident in the increasing number of clinical trials employing EV-based therapies (Table 2) [117].

Apart from their therapeutic value as drug delivery systems, exosomes can also be potentially utilized as biomarkers for clinically diagnostics of diseases. Owing to the wide range of DNA, RNA, and protein contents in EVs and that EVs are present in almost all body fluids, distinct molecular signatures (i.e., different combinations of RNAs and proteins) can be characterized from patients of a particular disease. These signatures can then be translated into useful diagnostic and prognostic information to identify subsets of susceptible individuals within the population. Indeed, numerous studies have since identified a wide array of exosomes carrying unique molecular signatures that may function as potential biomarkers for specific diseases, including cancer, and autoimmune and neurodegenerative diseases [118–121]. Importantly, these properties may allow EVs to be harnessed for precision medicine. The distinct molecular signatures of EVs characterized from different body fluids of a patient can be further assembled into a ‘personalized’ library to facilitate the identification of diagnostic markers and therapeutic targets that are specific to his profile. Furthermore, the comprehensive identification of EV surface markers and EV sources may enable the potential use of EVs as tissue or cell-specific delivery vectors for therapies.

## 7. Future Perspectives

With the growing evidence to support the functional links between EVs and diseases, as well as emerging capabilities of EVs as attractive diagnostic and therapeutic tools, it is imperative to gain full insights into the characteristics and regulation of EV uptake. Advancements in our knowledge of EV uptake specificity will no doubt aid in the development of novel therapeutic strategies to inhibit the interactions between disease-causing EVs and recipient cells, as well as to allow for the engineering of effective drug delivery systems. Along with the continuous improvements in EV isolation and characterization methods to address EV heterogeneity, understanding EV internalization will increase our ability to fully leverage the enormous potential of EVs and facilitate their translation from bench to bedside.

## Figures and Tables

**Table 1.** Examples of pathological diseases associated with EV cargo

Disease	EV Sources	EV Cargo	Potential Functions of EV Cargo	References
Glioblastoma (GBM)	Apoptotic GBM cells	Splicing factor RBM11	Increased proliferation and therapeutic resistance	[88]
Lung and breast cancer	Lung and breast cancer cells	miR-23a, miR-96, miR-105 and small nucleolar RNAs (snRNAs)	Enhanced angiogenesis; Immuno-modulation	[89–91]
Breast cancer	Breast cancer cells	miR-122	Reprogramming metabolism	[92]
Pancreatic cancer	Pancreatic ductal adenocarcinomas cell lines	Macrophage migration inhibitory factor (MIF)	Increased liver metastasis	[93]
Atherosclerosis	Human coronary endothelial cells and neutrophils	miR-155 and adhesion proteins	Increased inflammation and monocyte infiltration into plaques	[94,95]
Prion disease	Mouse plasma and neuroglial cells	Prion protein isoform PrP^SC^	Accumulation of infectious PrP^SC^	[96,97]
Alzheimer’s disease (AD)	Human and mouse primaryastrocytes	Amyloid-β (Aβ) and hyperphosphorylated Tau (p-Tau)	Aggregation of Aβ and p-Tau plaques	[98,99]
Parkinson’s disease (PD)	Human neuroglioma cells, mouse primary neurons	α-synuclein	Accumulation of toxic α-synuclein oligomers	[100,101]

**Table 2.** Examples of ongoing clinical trials involving EV-based therapies

Disease	EV Source	EV Modification	Phase, Cohort	NIH Clinical Trial Identifier
Acute ischemic stroke	Mesenchymal stromal cells (MSCs)	Enriched with miR-124	Phase 1/2, N = 5	NCT03384433
Bronchopulmonary dysplasia	MSCs	Not specified	Phase 1, N = 18	NCT03857841
Colon cancer	Plant	Loaded with curcumin	Phase 1, N = 35	NCT01294072
Malignant ascites and pleural effusion	Tumor-derived	Loaded with chemotherapeutic drugs	Phase 2, N = 30	NCT01854866
Malignant pleural effusion	Malignant pleural effusion	Loaded with methotrexate	Phase 2, N = 90	NCT02657460
Metastatic pancreatic cancer	MSCs	KrasG12D siRNA	Phase 1, N = 28	NCT03608631
Macular holes (MHs)	MSCs	Not specified	Phase 1, N = 44	NCT03437759
Radiation and chemotherapy-induced oral mucositis	Grape-derived	Unmodified	Phase 1, N = 60	NCT01668849
Ulcers	Plasma	Unmodified	Phase 1, N = 5	NCT02565264
